# Blending Powder Process for Recycling Sintered Nd-Fe-B Magnets

**DOI:** 10.3390/ma13143049

**Published:** 2020-07-08

**Authors:** Pavel A. Prokofev, Natalia B. Kolchugina, Katerina Skotnicova, Gennady S. Burkhanov, Miroslav Kursa, Mark V. Zheleznyi, Nikolay A. Dormidontov, Tomas Cegan, Anna S. Bakulina, Yurii S. Koshkidko, Bedřich Smetana

**Affiliations:** 1 Baikov Institute of Metallurgy and Materials Science, Russian Academy of Sciences, 119334 Moscow, Russia; nkolchugina@imet.ac.ru (N.B.K.); gburkhanov@imet.ac.ru (G.S.B.); markiron@mail.ru (M.V.Z.); ontip@mail.ru (N.A.D.); annbak@mail.ru (A.S.B.); 2 Joint Stock Company “Spetsmagnit”, 127238 Moscow, Russia; 3 Faculty of Materials Science and Technology, VSB—Technical University of Ostrava, 70800 Ostrava, Czech Republic; katerina.skotnicova@vsb.cz (K.S.); miroslav.kursa@vsb.cz (M.K.); tomas.cegan@vsb.cz (T.C.); bedrich.smetana@vsb.cz (B.S.); 4 Institute of New Materials and Nanotechnologies, National University of Science and Technology, 119991 Moscow, Russia; 5 Institute of Low Temperature and Structure Research, Polish Academy of Sciences, 50–422 Wroclaw, Poland; y.koshkidko@intibs.pl

**Keywords:** Nd-Fe-B magnets, recycling, grain-boundary diffusion, hydrogen decrepitation, coercive force, remanence

## Abstract

The wide application of Nd-Fe-B permanent magnets, in addition to rare-earth metal resource constraints, creates the necessity of the development of efficient technologies for recycling sintered Nd-Fe-B permanent magnets. In the present study, a magnet-to-magnet recycling process is considered. As starting materials, magnets of different grades were used, which were processed by hydrogen decrepitation and blending the powder with NdH_x_. Composition inhomogeneity in the Nd_2_Fe_14_B-based magnetic phase grains in the recycled magnets and the existence of a core-shell structure consisting of a Nd-rich (Dy-depleted) core and Nd-depleted (Dy-enriched) shell are demonstrated. The formation of this structure results from the grain boundary diffusion process of Dy that occurs during the sintering of magnets prepared from a mixture of Dy-free (N42) and Dy-containing magnets. The increase in the coercive force of the N42 magnet was shown to be 52%. The simultaneous retention of the remanence, and even its increase, were observed and explained by the improved isolation of the main magnetic phase grains as well as their alignment.

## 1. Introduction

The demand for rare-earth permanent magnets continues its strong and steady growth, due to their application in existing and future energy systems. Therefore, the rare-earth metals (REMs) are among the most critical elements, in particular, from the viewpoint of their availability. This fact determines the problem of the development of efficient technologies for recycling sintered Nd-Fe-B permanent magnets, which is closely related to the development of new approaches to the formation of high-coercivity and high-performance states of the Nd-Fe-B permanent magnet materials. One approach to solving the problem consists of using the grain boundary modification (GBM) of sintered magnet materials [1,2], which includes grain-boundary diffusion (GBD) and grain-boundary structuring (GBS). These processes effectively increase the coercivity of Nd-Fe-B magnets with a small amount of rare earth additives; the remanence is usually only slightly reduced [3]. At present, these processes are realized in the course of the recycling process of sintered Nd-Fe-B magnets, are unable to substantially change (melt or decompose) the Nd_2_Fe_14_B-based phase, and allow one to closely tailor the properties of magnets to meet a wide variety of end-user applications. The considered GBM approach is used for so-called magnet-to-magnet recycling, which assumes that the metals in sintered Nd-Fe-B magnets are recycled simultaneously [4]. GBM can be realized via the careful addition of compounds or blended elements and the application of hydrogen [5].

The application of hydrogen decrepitation (HD) as a process for recycling Nd-Fe-B sintered magnets was reported in [6], in which the essential role of hydrogen in the recycling process was demonstrated. The obtained magnetic properties of the recycled magnet were (*BH*)_max_ = 290 kJ/m^3^ (±5 kJ/m^3^), Br = 1240 mT (±50 mT) and _j_*H*_c_ = 830 kA/m (±50 kA/m); the decreases in the properties were15%, 10% and 20%, respectively, with respect to the properties of the initial magnet. The obtained properties in [2] were (*BH*)_max_ of 391 kJ/m^3^, *B*_r_ of 1.423 T and _j_*H*_c_ of 1041 kA/m.

REM hydrides may be used in grain-boundary modifications of sintered magnets [5]. In [7], additional blending with a fine powder of neodymium hydride was applied after the first milling during recycling. It was shown that the addition of 1 at.% of neodymium hydride was sufficient to maintain the density and magnetic properties of the recycled magnets. In [1,8], the recycling of waste sintered Nd-Fe-B permanent magnets by doping DyH_3_ nanoparticles is reported. As the content of DyH_3_ nanoparticles increased, the coercivity of recycled magnet increased gradually. The recycled magnets with DyH_3_ nanoparticle content between 0.0 and 1.0 wt.% maintained remanence (*B*_r_), but with higher additions, this began to decrease rapidly. The best recycled magnet produced contained 1.0 wt.% DyH_3_ nanoparticles; its properties, when compared those of the starting waste sintering magnet, were _j_*H*_c_, *B*_r_, and (*BH*)_max_ values of 101.7%, 95.4%, and 88.58%, respectively.

Hard disk drivers (HDDs) are one source of sintered Nd-Fe-B magnets that can be recycled; HDDs have been identified as an abundant, readily available source of scrap. In [9,10], hydrogen was used as a processing agent to decrepitate the sintered magnets contained in HDDs. It was shown in [9,10] that hydrogen is a very effective agent for the extraction of Nd-Fe-B magnets from HDDs; moreover, this technique can also be applied to other devices such as electric motors, generators and actuators. By concentrating the extracted materials by using further sieving and mechanical separation steps, it is possible to reduce the quantities of contaminants to a level whereby the extracted Nd-Fe-B powder can be used directly to form new magnetic materials. Moreover, the nickel coating peels away from the surface.

In [1], the addition of a (Nd_0.22_Dy_0.78_)(Co_0.84_Cu_0.12_Fe_0.04_)_0.84_ alloy was used in a recycling process which yields magnets that are suitable only for room temperature applications. The recycled magnets were transformed into high coercivity magnets via controlled elemental addition. However, the addition resulted in a decrease in remanence values.

The aim of the present study is to investigate the possibility of recycling Nd-Fe-B magnets using hydrogen decrepitation, a blending powder procedure and mixtures of magnets of different grades.

## 2. Materials and Methods

As the initial magnets, we used N42 grade magnets (separated from electronic devices), the magnetic characteristics of which were *B*_r_ = 1.31 T, _j_*H*_c_ = 1093 kA/m, (*BH*)_max_ = 336 kJ/m^3^, and ill-conditioned magnets having the following composition: (wt.%) Nd-20.7, Pr-5.6, Dy-6.6, B-1, Cu-0.22, Al-0.53, Co-0.3, Fe-bal. The late magnets were intended for high coercivity assemblies; the magnet characteristics were *B*_r_ = 1.13 T, _j_*H*_c_ = 2150 kA/m, (*BH*)_max_ = 250 kJ/m^3^, which corresponds to the grade of N33U. The preparation of the feedstock, in which the proportion Dy-free-to-Dy-containing magnets was 3:1, consisted of the preliminary demagnetization and mechanical crushing of magnets, which effected deep hydrogenation accompanied by the exfoliation of the Ni-based protective coating. The feedstock in the form of crushed Nd-Fe-B-based magnets was subjected to hydrogen decrepitation at 250 °C in hydrogen flow at a pressure of ~100kPa. Neodymium hydride NdH_~2_ was prepared by the hydrogenation of Nd under the conditions used for the decrepitation of Nd-Fe-B magnets. The powder prepared as a mixture of decrepitated magnets of two grades was passed through a sieve in order to separate particles of the Ni-based coating, which was peeled from the magnets and the Nd-Fe-B-based powder. The powder mixture and neodymium hydride added before the fine milling stage were milled in a vibratory ball mill in an isopropyl alcohol medium. The neodymium hydride was added to increase the physical density of the powder blanks. After fine milling, the wet powder mixture (in isopropyl alcohol) was compacted in a transverse magnetic field of no less than 1540 kA/m.

The phase composition of the powder after hydrogen decrepitation was studied using a (N42 + NdH_2_) powder mixture. X-ray diffraction analysis was performed on an Ultima IV diffractometer (“Rigaku”, Tokyo, Japan) equipped with a “D/teX” detector; Co*K*α radiation and a scanning step of 0.001° were used. The compacted magnet blanks were sintered at ~1118 °C for 2 h. The subsequent heat treatment included heating to 500 °C and holding at this temperature for 1 h, followed by quenching with gaseous nitrogen. A Quanta 450 FEG high-resolution field emission gun scanning electron microscope (FEI Company, Fremont, CA, USA) equipped with an energy-dispersive spectroscopy (EDS, EDAX Inc., Mahwah, NJ, USA) microprobe was used to investigate the structure, chemical composition and distribution of the magnet components (X-ray mapping). The hysteretic properties of the magnet were measured at room temperature in a magnetic field of 2500 kA/m using an MH-50 automatic hysteresisgraph system (Walker Scientific Inc., Worcester, MA, USA). To understand the processes that occurred during sintering, and to determine the heat treatment conditions, a differential thermal analysis (DTA) was performed under an argon atmosphere at a heating rate of 30 °C/min using a Setsys−1750 installation (Setaram Instrumentation, Caluire, France).

## 3. Results and Discussion

### 3.1. X-ray Diffraction Studies

**Figure 1:**
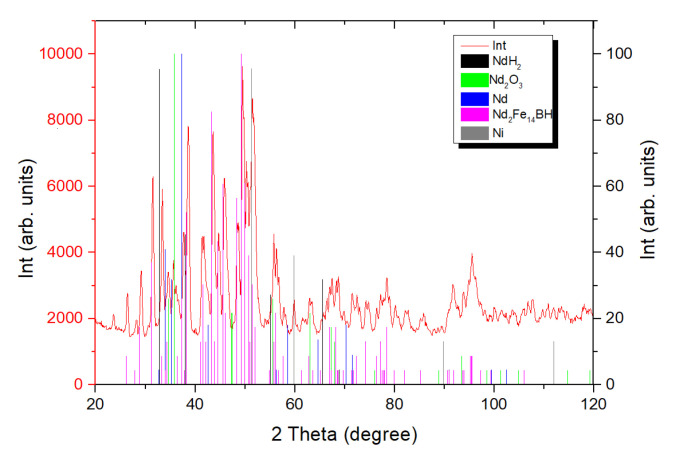
X-ray diffraction pattern of the powder mixture obtained after decrepitation of an N42 magnet and its milling with +2 wt.% NdH_2_.

Figure 1 shows the X-ray diffraction pattern of powder prepared by the hydrogen decrepitation of an N42 grade magnet and subsequent milling with neodymium hydride (2 wt.%). The phase composition of the powder mixture and the lattice parameters of the found phases are given in Table 1.

The identified phases are typical of powder mixtures obtained after hydrogen decrepitation. Therefore, it was assumed that the hydrogenation of the Dy-containing magnet would result in the formation of a hydrogenated, Dy-containing main magnetic phase, (Nd,Dy), likely (Nd,Dy)H_x_. REM oxides and nickel can also be present. In the case of the recycling process, a small amount of Ni originating from the initial magnet coating was present. It should be noted that a high nickel content in the powder mixture can lead to a decrease in the physical density of the prepared magnets, worsening their magnetic hysteretic characteristics (in particular, the coercive force), as well as the formation of laminations on the magnet surface. The presence of Ni in Nd-Fe-B-based powders manufactured from hard-disk devices was demonstrated in [10,11]. Sieving, combined with mechanical agitation, decreases the Ni content to ~325 ppm [10]. When the Ni-containing coating was removed prior the preparation of the powder, the Ni content in the final magnet was 300 ppm. This fact indicates the possibility of the removal of the coating by sieving after hydrogen decrepitation, since no significant difference in its content was observed between the two methods of Ni removal during recycling.

The hydrogenated NdFeB powder already had a fine, aligned microstructure, retained from the starting material, and therefore, much less milling was required than in the case of the cast and subsequently hydrogenated Nd-Fe-B-based alloy.

### 3.2. Microstructural Characteristics

**Figure 2:**
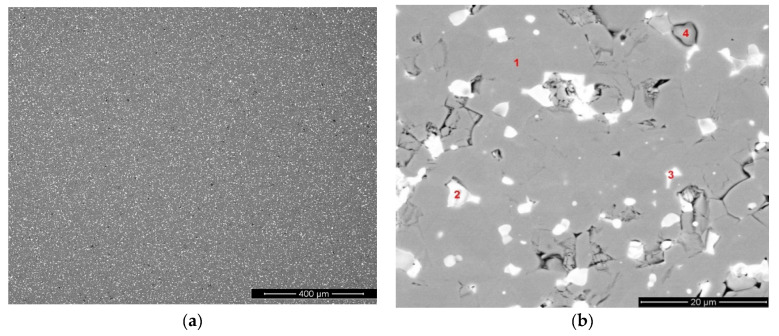
(**a**,**b**) Microstructure (SEM image, BSED) of Nd-Pr-Dy-Fe-B magnet prepared by recycling.

The structure of sintered magnets prepared by recycling was studied by electron scanning microscopy and EDX analysis. The microstructure of the magnet is given in Figure 2a,b. The chemical compositions of the observed phases are given in Table 2.

According to the data, phase 1 corresponded to the main magnetic (Nd,Dy)_2_Fe_14_B phase. The REM-rich phases (phases 2 and 3) differed in the Nd, Pr and Dy contents. Phase 2 is likely to be inherited from the Dy-containing starting magnet; the high Pr and Dy contents in the phase seem to support this assumption. The high Pr content in phase 3 also demonstrated the trend of Pr presence in the intergranular phase when conditions for grain-boundary diffusion were applied.

Phase 4 was an oxide based on Nd and containing also Dy and Pr. It should be noted that the Pr content in the oxide phase was higher than that of Dy; this fact was related to the higher oxidizing ability of the light rare-earth. Nickel was also present within the grain-boundary phases.

**Figure 3:**
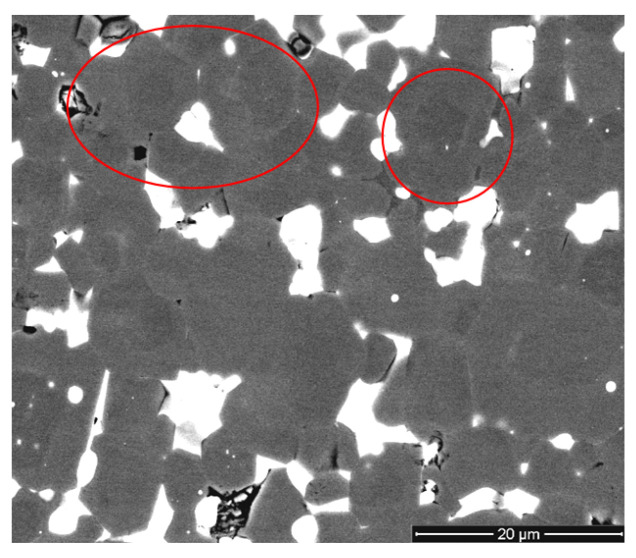
Microstructure (SEM image, BSED, improved contrast) of the magnet prepared by recycling magnets of two different grades.

Figure 3 shows the microstructure of the magnet, which is presented with slightly improved contrast. There were distinct (Nd,R)_2_Fe_14_B (R = Dy, Pr) core-shell microstructures and composition nonuniformity among the main magnetic-phase grains, as indicated by the obvious bright and dark gray contrasts (shown by red circles in Figure 3).

**Figure 4:**
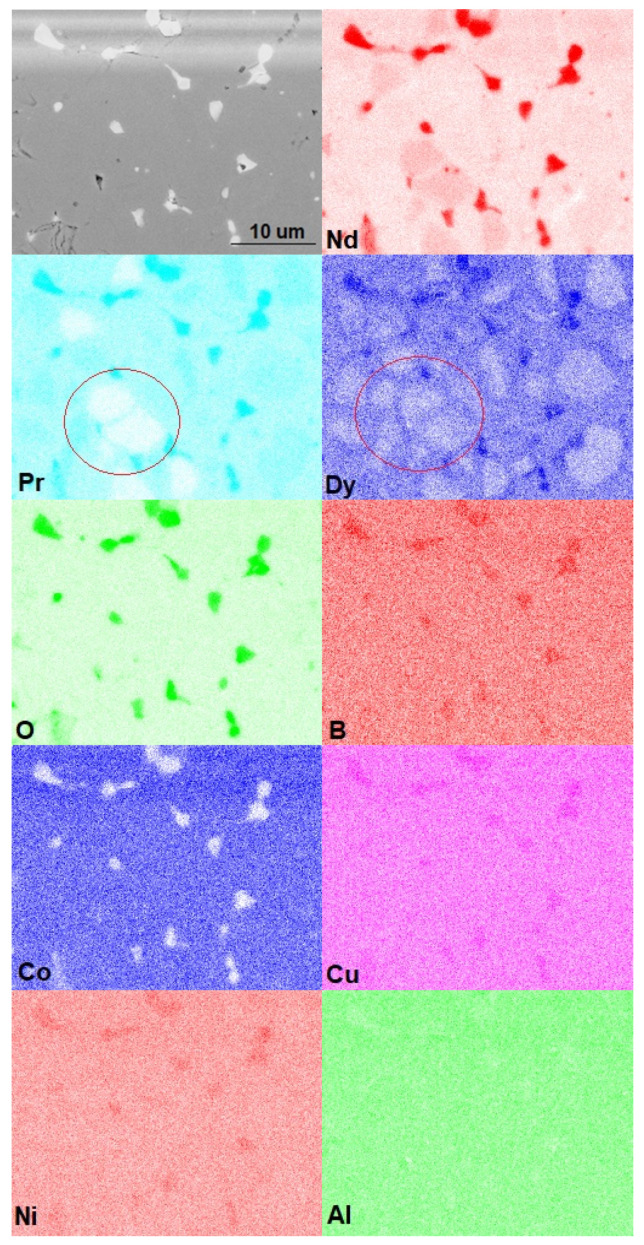
SEM image and elemental mapping of Nd, Pr, Dy, O, B, Co, Cu, Ni and Al.

To clarify the distributions of elements in the phases and within the grains of the main magnetic (Nd,Dy)_2_Fe_14_B phase, X-ray elemental mapping was performed (Figure 4). As shown, grains enriched in Nd and depleted of Pr were observed (as shown by the red circles in Figure 4), which were inherited from magnets of different grades. The dysprosium distribution demonstrated obvious core-shell structures; Dy, along with the other REMs, was also present in triple junctions of the 2-14-1 phase grains. The triple junctions were enriched in oxygen; this indicated the presence of Nd, Dy, and Pr in the form of oxides. Cobalt was present in all phases; this fact indicated the formation of (Nd,Dy)_2_(Fe,Co)_14_B with dissolved Co. Nickel was also observed in the triple junctions and oxide phases.

It is well known that the effect of Cu addition to Nd-Fe-B sintered magnets is the improvement of wettability of the Nd-rich phase, leading to the homogeneous formation of a Nd-rich liquid during sintering. Usually, after sintering, copper, as a component of a low-melting eutectic, is present at triple junctions of Nd_2_Fe_14_B-based grains. Copper, being a component of the initial magnets used in the recycling process, was also present at the triple junctions in the recycled magnet.

The formation of a typical core-shell structure of (Nd,Dy)_2_Fe_14_B, which has a higher anisotropy field compared to that of Nd_2_Fe_14_B [11], is the key to increasing the coercivity of Nd-Fe-B magnets by diffusing heavy rare earth elements.

The formation mechanism of the core-shell structure in the magnets prepared from the powder mixture containing Nd_2_Fe_14_BH_x_, (Nd,Pr,Dy)_2_Fe_14_BH_x_, Nd, NdH_x_ and small amounts of Dy and DyH_x_ is as follows. During sintering, when the compacted magnet blank was heated to the sintering temperature, Nd-rich + (Nd,Dy,Pr)-rich liquid was formed. Small amounts of the Nd_2_Fe_14_B and (Nd,Pr,Dy)_2_Fe_14_B matrix phases melted because of the ternary eutectic reaction that occurred when the temperature was higher than 680 °C [12]; Dy atoms present in the liquid diffused into the Nd_2_Fe_14_B-based phase, leading to the formation of (Nd,Dy)_2_Fe_14_B core-shell microstructures after cooling. This mechanism commonly occurs during the formation of core-shell structures. However, in the case of the mixture of Dy-containing and Dy-free powders, the dysprosium content in the liquid was too small to form the Dy shells observed in Figure 3 and Figure 4d. Therefore, we can assume that the solid-phase diffusion of Dy also occurred at the contact area of Dy-free and Dy-containing grains in the absence of the intergranular phase. Solid-phase diffusion occurred at the expense of the Dy concentration gradient. In the case of solid-phase diffusion, lattice diffusion of Dy atoms took place; the observed Dy-rich shells were sufficiently thin. During lattice diffusion, Dy atoms partially substituted Nd atoms in the Nd_2_Fe_14_B phases to form (Nd, Dy)_2_Fe_14_B shells, and the Nd atoms were repelled to the grain-boundary phases during the process. This results in the formation of thicker grain-boundaries (in the case of liquid-phase diffusion) with a higher Nd concentration, and in the formation of a thin, Nd-rich boundary (in the case of solid-phase diffusion). In both cases, the magnetic isolation of the grains improved.

As shown in [13], the Ni was always in the GB phases. The presence of Al and Ni in the GB phases reduced the melting point of the grain boundary phases and increased the diffusion coefficient of Dy in the grain boundaries.

### 3.3. Study of Magnetic Properties of Magnets Prepared by Recycling

**Figure 5:**
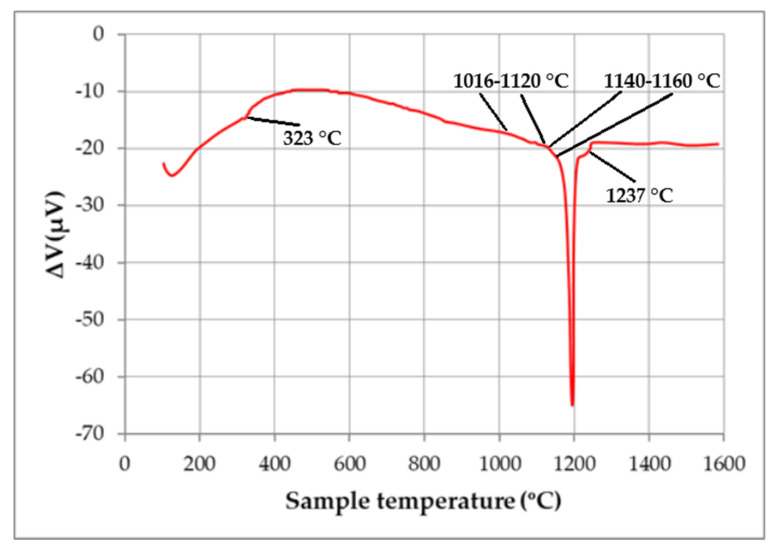
DTA curve measured during heating of the magnet prepared by recycling.

The sintering temperature of the magnets was corrected using DTA data (Figure 5). A number of anomalies which occurred within a temperature range of 1080–1120 °C allowed us to accurately choose the sintering temperature of the magnets, which was 1118 °C; the temperature of post-sintering heat treatment was 500 °C (1 h). The Curie temperature of these magnets was determined to be equal to 323 °C (see Figure 5).

**Figure 6:**
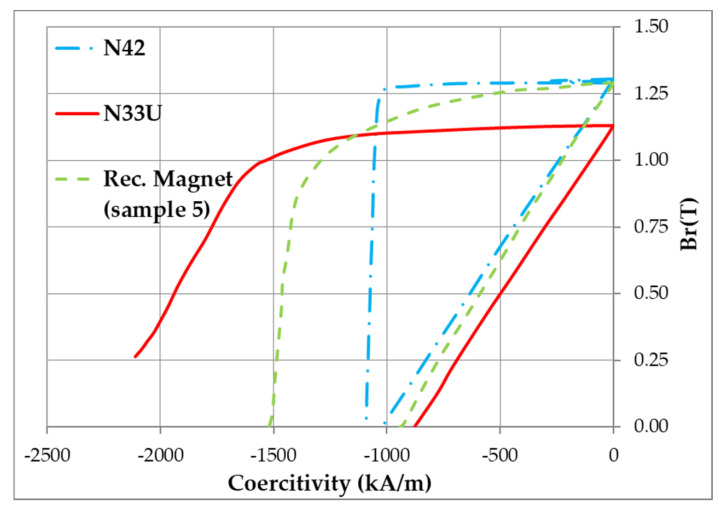
Magnetization reversal curve of recycled magnet (green line, sample 5 in Table 3) in comparison with those of the initial N42 (blue line) and initial high-coercivity N33U (red line) magnets.

The magnetic characteristics of the magnets prepared using N42 and high-coercivity Dy-containing magnets are given in Table 3 and Figure 6. For comparison, Table 3 shows the magnetic characteristics of magnets prepared by the recycling process using scraps of magnets and HDD magnets taken in different proportions.

The data given in Figure 6 and Table 3 indicate that the recycling process allowed us to prepare magnets with a coercive force exceeding that of an N42 magnet; this was related to the existence of a core-shell structure of Nd_2_Fe_14_B-based grains (Figure 4 and Figure 5d) at the expense of the Dy present in the second component of the mixture (magnet N33U). Moreover, an increase in the remanence of the recycled magnet, compared to that of an N42 magnet, was observed (Table 3). This was related to the use of NdH_x_ in the powder mixture. According to the data given in [14,15], in which NdH_x_ was added to the powder mixture, the cause for the unique simultaneous enhancement of remanence and coercive force was attributable to the isolation of the magnetic coupling between Nd_2_Fe_14_B-based grains by creating nonmagnetic Nd-rich grain boundaries, and the enhanced alignment of the Nd_2_Fe_14_B-based hard magnetic phase, which was fabricated under optimal diffusion conditions.

## 4. Conclusions

Grain boundary diffusion engineering is a revolutionary production technology of Nd-Fe-B magnets, which can be used in the recycling process, since it effectively ensures the utilization of rare earth elements from waste magnets and saves rare earth resources.

The recycling process of N42 and N33U (Dy-containing) magnets (magnet-to-magnet recycling) improves the coercivity of N42 magnets by using reduced contents of heavy rare-earth metal. It was demonstrated that the existence of the core-shell structure in the recycled magnet ensures the improvement of the coercivity. Usually, the coercivity enhancement by Dy substitution is achieved at the expense of a decrease in the remanence, and therefore, in the maximum energy product because of the antiferromagnetic coupling of Dy and Fe atoms in the (Nd, Dy)_2_Fe_14_B lattice [12].

In the present study, a simultaneous increase in the remanence and coercive force was observed, which was related to the improved isolation of the main magnetic phase grains, and probably also to the improvement of their alignment. This was due to the use of a hydride (NdH_x_) of light rare-earth metals in the powder mixture, which was mainly distributed within the grain boundaries of the magnets, and which regulated the grain boundary phases and increased the fraction of the grain boundary Nd-rich phases to ensure their homogeneity.

## Figures and Tables

**Table 1:** Phase composition of the powder mixture obtained after decrepitation of an N42 magnet and its milling with +2 wt.% NdH_2_.

Phase	Content (wt.%)	Space Group	Lattice Parameters (nm)		
			a	b	c
Nd_2_Fe_14_BH_1.04_	78	P4_2_/mnm	0.88527(18)	0.88527(18)	1.2277(3)
NdH_2_	9	Fm-3m	0.54337(7)	0.54337(7)	0.54337(7)
Nd_2_O_3_	4	P-3m_1_	0.3758(5)	0.3758(5)	0.6306(11)
Nd	6	P6_3_/mmc	0.3565(5)	0.3565(5)	1.211(2)
γNi	3	Fm-3m	0.36079(17)	0.36079(17)	0.36079(17)

**Table 2:** Chemical compositions (at.%) of phases present in the recycled Nd-Pr-Dy-Fe-B magnet.

Element/Phase	O	Dy	Al	Pr	Nd	Fe	Co	Ni	Cu
Phase_1		0.8	0.8	1.5	11.8	83.6	1.5	0.0	0.0
Phase_2		8.6	0.0	10.8	58.3	21.3	0.6	0.3	0.0
Phase_3		1.2	1.5	6.9	31.8	54.8	1.8	0.5	1.4
Phase_4	61.4	2.1	0.2	3.7	20.9	11.2	0.3	0.1	0.0

**Table 3:** Magnetic properties of Nd-Fe-B-based magnets prepared by recycling.

Sample No.	Magnet Feedstock + 2% NdH_x_	*B*_r_	_b_ *H*_c_	_j_H_c_	(*BH*)_max_
		(T)	(kA/m)	(kA/m)	(kJ/m^3^)
	Starting N42	1.31	1020	1093	336
	Starting N33U	1.13	880	2150	250
1	3 kg N42 + 1 kg N33U	1.39	1014	1368	368
2	3 kg N42 + 1 kg N33U	1.32	949	1457	324
3	3 kg N42 + 1 kg N33U	1.32	970	1663	335
4	3 kg N42 + 1 kg N33U	1.41	1009	1360	370
5	3 kg N42 + 1 kg N33U	1.29	938	1514	314
6	3 kg N42 + 1 kg N33U	1.39	1026	1340	379
7	3 kg waste of N42 + 1 kg magnets from HDD	1.41	878	902	383
8	3 kg waste of N42 + 1 kg magnets from HDD	1.39	1015	1128	372
9	2 kg waste of N42 + 1 kg magnets from HDD + 1 kg N33U	1.32	978	1413	341
